# Microwave-Assisted Expeditious Synthesis of 2-Alkyl-2-(*N*-arylsulfonylindol-3-yl)-3-*N*-acyl-5-aryl-1,3,4-oxadiazolines Catalyzed by HgCl_2_ under Solvent-Free Conditions as Potential Anti-HIV-1 Agents

**DOI:** 10.3390/molecules23112936

**Published:** 2018-11-10

**Authors:** Zhiping Che, Yuee Tian, Shengming Liu, Jia Jiang, Mei Hu, Genqiang Chen

**Affiliations:** Laboratory of Pharmaceutical Design & Synthesis, Department of Plant Protection, College of Forestry, Henan University of Science and Technology, Luoyang 471003, China; tianyuee1985@163.com (Y.T.); zhenxiziji2005@126.com (S.L.); lxy_jiangjia@126.com (J.J.); lxy_humei@163.com (M.H.); genqiangchen@126.com (G.C.)

**Keywords:** 1,3,4-oxadiazolines, microwave irradiation, HgCl_2_ catalyst, anti-HIV-1 activity

## Abstract

A series of 2-alkyl-2-(*N*-arylsulfonylindol-3-yl)-3-*N*-acyl-5-aryl-1,3,4-oxadiazolines were expeditious prepared under microwave-assisted, catalyzed by HgCl_2_ and solvent-free conditions. This method has the advantage of low catalyst loading and recovering catalyst, ease reaction and repaid reaction times, easy separation products and excellent yields, and more conducive to the large-scale synthesis products. Furthermore, compounds **3s**, **3y**, **3a′**, **3b′**, **3f′**, **3i′**, **3q′**, and **3r′** exhibited more potent anti-HIV-1 activity with EC_50_ values of 3.35, 6.12, 3.63, 9.54, 1.79, 0.51, 3.00, and 4.01 μg/mL, and TI values of 32.66, >32.68, 31.22, 13.94, 24.27, 39.59, 26.01, and 24.51, respectively. Especially compound **3i′** displayed the highest anti-HIV-1 activity with TI values of 39.59.

## 1. Introduction

Acquired immunodeficiency syndrome (AIDS) is mainly caused by human immunodeficiency virus type 1 (HIV-1) infection and has remained one of the most difficult medical barriers for human health since it was first reported in 1981 [1]. The reverse transcriptase (RT) of the HIV-1 plays a significant role in the viral replication process, which makes it a pivotal target for anti-HIV-1 inhibitor discovery [2,3]. Although numerous RT inhibitors, including primarily the nucleoside/nucleotide RT inhibitors (NRTIs) and non-nucleoside RT inhibitors (NNRTIs), have been developed, like other anti-HIV inhibitors, effectiveness of now approved NRTIs and NNRTIs have been hampered because of the fast development of resistance [4,5,6,7]. It is estimated that 36.9 million people (including 2.6 million children) were living with HIV infection in the year 2014 according to *UNAIDS*-2015 report, and 1.2 million people died due to HIV as well as related diseases [8]. To circumvent this challenge, it is urgent to discover and develop safe, green, efficient, selective, and novel anti-HIV inhibitors having significant potency against drug-resistant RT viral strains as well as less toxicity [8,9,10,11]. 

To the best of our knowledge, 1,3,4-oxadiazolines are excellent candidates for the research of anti-HIV-1 agents, antibacterial agents, antitumor agents, and chitin biosynthesis inhibitors [12,13,14,15]. As our ongoing work on research of anti-HIV-1 inhibitors, 2-alkyl-2-(*N*-arylsulfonylindol-3-yl)-3-*N*-acyl-5-aryl-1,3,4-oxadiazoline derivatives were further prepared through a modified route.

Generally, there are three ways to synthesize 1,3,4-oxadiazolines. The first kind is the traditional procedure, which is usually require excess of anhydride, and long reaction time [16,17,18]. The second is the ultrasonic irradiation assisted synthesis, it is with relatively mild reaction conditions and higher yield [19]. The third is the microwave irradiation assisted synthesis, which are under solvent-free conditions with short reaction time [15]. Although many advantages, there need to be further improved on the methods of the ultrasonic irradiation and microwave irradiation assisted synthesis of 1,3,4-oxadiazolines. On the other hand, since the first report of microwave irradiation assisted synthesis in 1986 [20,21], the technique has been accepted as a method for reducing reaction times and increasing yields of product compared to conventional procedure [22,23,24,25,26]. Especially, the use of microwave ovens as tools for synthetic chemistry is a fast growth area [27,28,29,30,31,32], so here we aimed to use this method to synthetize 2-alkyl-2-(*N*-arylsulfonylindol-3-yl)-3-*N*-acyl-5-aryl-1,3,4-oxadiazolines catalyzed by HgCl_2_ under solvent-free conditions.

## 2. Results and Discussion

### 2.1. Chemistry

To find out the most compatible reaction conditions for synthesizing 2-alkyl-2-(*N*-arylsulfonylindol-3-yl)-3-*N*-acyl-5-aryl-1,3,4-oxadiazolines, the reaction of *N*-benzenesulfonyl-3-acetylindole benzoyl hydrazone (**1a**) with acetic anhydride (**2a**) under microwave irradiation and solvent-free conditions was investigated, and a wide range of reaction catalysts were also tested. As can be clearly seen in Table 1, the cyclization reaction catalyzed by MgCl_2_^.^6H_2_O as a Lewis acid catalyst was found to be sluggish at best condition, providing **3a** in 38% yield and recycling **1a** in 37% yield after 10 × 3 min following purification by preparative thin-layer chromatography (entry 1). The reaction was accelerated dramatically by the presence of ZnCl_2_ providing the product **3a** in 48% yield and recycling the raw material **1a** in 23% yield after 10 × 3 min (entry 2). Although these experiments were not so efficient, the use of solvent-free reaction conditions does have some intrinsic ecological and chemical value. Inspired by these results, Lewis acid catalyst (entry 3) was found to be greatly enhancing the reaction in AlCl_3_ catalyzed, which providing **3a** in 53% yield after 10 × 3 min following purification on silica. Similarly, reactions were improved by the presence of SnCl_2_^.^2H_2_O (entry 4) and FeCl_3_ (entry 5) providing the product **3a** in 63% and 68% yield, respectively. However, the optimum conditions for this transformation employed HgCl_2_ as a Lewis acid catalyst, providing **3a** in 91% yield after 10 × 3 min (entry 6). 

In order to further optimize the reaction conditions, a wide range of reaction parameters were tested by altering the amount of **2a** and catalyst as well as the reaction time in a test reaction of **1a** and **2a** (Table 2). 

As can be clearly seen in Table 2, when 0.5 mmol of **1a** and 2.5 mmol of **2a** reacted by HgCl_2_ catalyst at the amount of 5 mol% under microwave irradiation, **3a** was obtained in a 97% yield after 10 × 2 min (entry 1). When the amount of **2a** reduced to 2.0 mmol, **3a** was obtained in a 90% yield for 10 × 2 min (entry 2). Moreover, when the amount of **2a** decreased to 1.5 mmol, **3a** was obtained in a 91% yield if the reaction time was prolonged to 10 × 3 min (entry 3). Nevertheless, if we keep reducing the amount of **2a** to 1.0 mmol, only providing **3a** in 41% yield and recycling **1a** in 55% yield after 10 × 4 min following purification by preparative thin-layer chromatography (entry 4). However, the cyclization reaction was terminated by the absence of HgCl_2_ even if the reaction time was prolonged to 10 × 12 min and the amount of **2a** was increased to 2.5 mmol (entry 5). Inspired by these phenomena, we realized that the amount of HgCl_2_ as a Lewis acid catalyst was significant for enhancing the cyclization reaction. For example, when 0.5 mmol of **1a** and 2.5 mmol of **2a** reacted by HgCl_2_ catalyst at the amount of 2.5 mol% under microwave irradiation, **3a** was synthesized in a 69% yield after 10 × 5 min (entry 6). When the amount of HgCl_2_ increased to 3.0 mol%, **3a** was prepared in a 78% yield for 10 × 3 min (entry 7). To our delight, when the amount of HgCl_2_ increased to 4.0 mol%, the cyclization reaction was accelerated dramatically providing the product **3a** in 91% yield after 10 × 3 min (entry 8). We concluded that when 0.5 mmol of **1a** and 1.5 mmol of **2a** reacted by HgCl_2_ catalyst at the amount of 4 mol% under microwave irradiation, **3a** was smoothly synthesized in a 90% yield after 10 × 3 min (entry 9).

Based upon the above results, a wide range of *N*-arylsulfonyl-3-acylindole arylcarbonyl hydrazones (**1**, R^1^ = H, 6-Me, 5-CN or 5-NO_2_; R^2^ = H, *p*-Me, *p*-Cl, *m*-NO_2_ or *p*-OMe; R^3^ = Me, Et or *n*-Pr; R^4^ = H or *m*-Me) with anhydrides (**2**, R^5^ = Me or Et) was investigated to explore the scope of the reaction. As outlined in Table 3, 2-alkyl-2-(*N*-arylsulfonylindol-3-yl)-3-*N*-acyl-5-aryl-1,3,4-oxadiazolines (**3a**–**d′**) were prepared in 80–95% yields for 10 × 3–10 × 4 min (Of course, the reaction can also be carried out directly in one go for 30 or 40 min, with little impact on yield). The steric and electronic effects of substituents of **1** to the reaction were not very obvious.

We have previously developed an efficient method for the synthesis of 2-monosubstituted 3-*N*-acyl-5-phenyl-1,3,4-oxadiazolines **3e′**–**r′** under ultrasonic irradiation [19], however, under the above reaction conditions, 2,2-disubstituted 3-*N*-acyl-5-phenyl-1,3,4-oxadiazolines were not obtained at all even if the reaction time was prolonged. So we have previously described another a convenient, rapid, and high-yielding reaction for the synthesis of 2,2-disubstituted 3-*N*-acyl-5-phenyl-1,3,4-oxadiazolines **3a**–**w** under microwave irradiation and solvent-free conditions [15]. On the other hand, compared with the previous procedure, the present methodology has the advantages of low catalyst loading and recovering catalyst, short reaction and repaid reaction times, easy separation products, and increased reaction yields (except **3i**, **3j**, **3o**, **3r**, **3t**, **3u**, and **3w**), and more conducive to the large-scale synthesis products.

In order to further study the cyclization reaction, we have done the following study. As illustrated in Table 4, when the cyclization reaction of **1a** (3 mmol) with **2a** (9 mmol) was complete, the reaction mixture was cooled to room temperature. Then the mixture was filtered and the filter residue was washed with dichloromethane (3 × 10 mL). The HgCl_2_ catalyst was reused directly in the next reaction. For examples, when 3 mmol of **1a** and 9 mmol of **2a** reacted by HgCl_2_ catalyst at the amount of 4 mol% under microwave irradiation, **3a** was smoothly synthesized in 98% yield after 5 min for the first time (entry 1). When the reaction time prolonged to 6 min, **3a** was obtained in 98% yield for recovering HgCl_2_ catalyst was reused directly in the reaction (entry 2). Entries 3 and 4, **3a** was obtained in 97% and 98% yield, respectively. 

To our delight, in the case of reaction amplification, it was found that HgCl_2_ had better catalytic effect on the reaction. It may be that HgCl_2_ induces the cyclization reaction in which the turnover frequency is accelerated, and the yield of compound **3a** increased significantly.

### 2.2. Biological Activities

**Figure 1:**
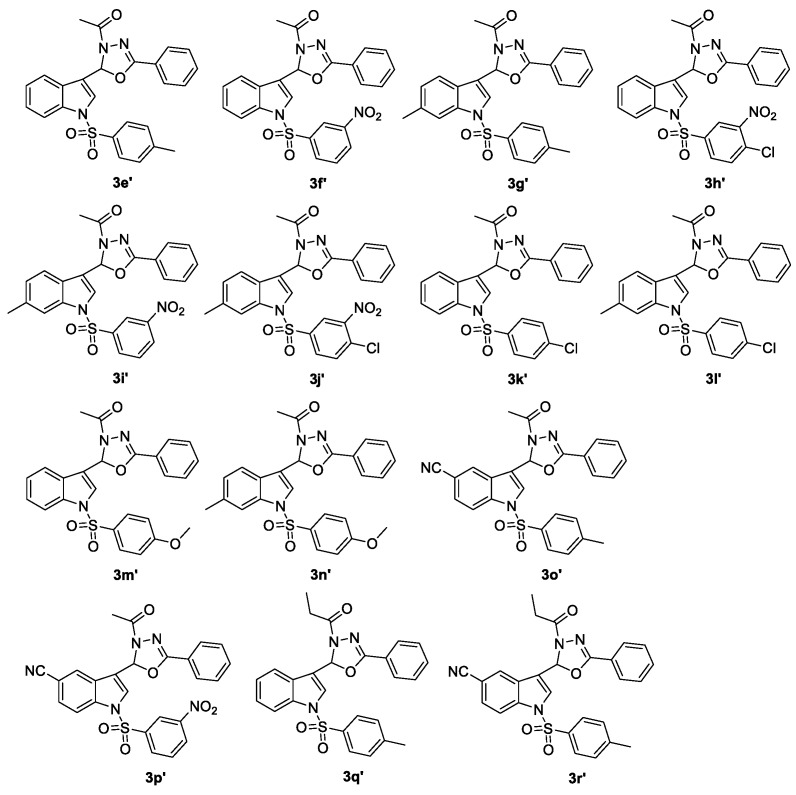
Chemical structures of 2-(*N*-arylsulfonylindol-3-yl)-3-*N*-acyl-5-aryl-1,3,4-oxadiazolines **3e′**–**r′**.

Fourteen compounds of 2-(*N*-arylsulfonylindol-3-yl)-3-*N*-acyl-5-aryl-1,3,4-oxadiazolines (**3e′**–**r′**, Figure 1) were prepared using our previously published method, and characterized by ^1^H-NMR, EI-MS or ESI-MS and HRMS [19]. Subsequently, 21 2-alkyl-2-(*N*-arylsulfonylindol-3-yl)-3-*N*-acyl-5-aryl-1,3,4-oxadiazolines (**3s**–**r′**) were tested in vitro for their anti-HIV-1 activity, and 3′-azido-3′-deoxythymidine (AZT) was used as a positive control illustrated in Table 5.

Among these tested 2-alkyl-2-(*N*-arylsulfonylindol-3-yl)-3-*N*-acyl-5-aryl-1,3,4-oxadiazolines, compounds **3s**, **3y**, **3a′**, **3b′**, **3f′**, **3i′**, **3q′**, and **3r′** exhibited more potent anti-HIV-1 activity with EC_50_ values of 3.35, 6.12, 3.63, 9.54, 1.79, 0.51, 3.00, and 4.01 μg/mL, and TI values of 32.66, >32.68, 31.22, 13.94, 24.27, 39.59, 26.01, and 24.51, respectively. Especially compound **3i′** displayed the highest anti-HIV-1 activity with TI values of 39.59. Meanwhile, a brief structure activity relationship (SAR) was determined. (1) When R^1^ = H or 6-Me, compound with R^2^ = *m*-NO_2_ was more vital for the anti-HIV-1 activities than R^2^ = *m*-NO_2_, *p*-Cl or *p*-Cl (**3f′** and **3i′** vs. **3h′** and **3j′**, EC_50_ values of 1.79, 0.51, 3.53, and 12.42 μg/mL, TI values of 24.27, 39.59, 1.36, and 0.18, respectively; that is, the TI value of **3f′** was close to 18 times of that of **3h′** and the TI value of **3i′** was close to 220 times of that of **3j′**. **3f′** and **3i′** vs. **3k′** and **3l′**; EC_50_ values of 1.79, 0.51, 19.37, and 57.49 μg/mL; TI values of 24.27, 39.59, 0.86, and 3.16, respectively; that is, the TI value of **3f′** was more than 28 times of that of **3k′** and the TI value of **3i′** was more than 12 times of that of **3l′**). (2) The length of the chain of substituent R^5^ was significant for the anti-HIV-1 activity (**3q′** vs. **3e′**, EC_50_ values of 3.00 and 15.52 μg/mL, TI values of 26.01 and 0.97, respectively, especially the TI value of **3q′** was close to 27 times of that of **3e′**; **3r′** vs. **3o′**, EC_50_ values of 4.01 and 18.14 μg/mL, and TI values of 24.51 and 8.36, respectively). (3) When R^1^ = 5-NO_2_, compound with R^2^ = H was more important for the anti-HIV-1 activities than R^2^ = *p*-Me or *p*-Cl (**3s** vs. **3b′** and **3c′**, EC_50_ values of 3.35, 9.54 and 6.01 μg/mL, TI values of 32.66, 13.94 and 8.89, respectively; that is, the TI value of **3s** was more than two times of that of **3b′** and was more than three times of that of **3c′**). When R^1^ = 5-CN, introduction of R^2^ as the *p-*Cl group could lead to the pronounced compound (**3a′** vs. **3z**, EC_50_ values of 3.63 and 17.57 μg/mL, TI values of 31.22 and 3.01, respectively, especially the TI value of **3a′** was more than 10 times of that of **3z**).

## 3. Experimental Section

### 3.1. General Information

All solvents and reagents were used as obtained from commercial sources without further purification. Analytical thin-layer chromatography (TLC) and preparative thin-layer chromatography (PTLC) were performed with silica gel plates using silica gel 60 GF_254_ (Qingdao Haiyang Chemical Co., Ltd., Shandong, China). Melting points are uncorrected. Nuclear magnetic resonance spectra (NMR) were recorded on a Bruker Avance DMX 400 MHz instrument (Bruker Daltonik, Bremen, Germany) in CDCl_3_ (^1^H at 400 MHz and ^13^C at 100 MHz) using TMS (tetramethylsilane) as the internal standard. Electrospray ion trap mass spectrometry (ESI-TRAP-MS) was carried out with a Bruker ESI-TRAP Esquire 6000 plus mass spectrometry instrument (Bruker, Germany) Microwave irradiation was performed in a Galanz microwave oven, WG700CTL20II-K6 (Galanz, Guangdong, China).

### 3.2. Preparation of 2-Alkyl-2-(N-arylsulfonylindol-3-yl)-3-N-acyl-5-aryl-1,3,4-oxadiazolines (***3a**–**d′***)

A mixture of **1** (0.5 mmol), **2** (1.5 mmol), and HgCl_2_ (0.02 mmol) was reacted under microwave heating at 700 W for 30–40 min. After allowing the mixture to cool to room temperature, the mixture was diluted with dichloromethane (30 mL) and filtered. The filtrate was washed with saturation sodium bicarbonate (2 × 20 mL), and brine (1 × 10 mL). Then the organic phase was dried over anhydrous Na_2_SO_4_, concentrated in vacuo and purified by preparative thin-layer chromatography (PTLC) to give the desired products **3a**–**d′** in 80–95% yields. Compounds **3a**–**w** were known compounds and characterized by comparison of the data as described in our previous paper [15]. The NMRs, MS (see Supplementary Materials) and typical spectral data of compounds **3s**–**d′** were as follows. 

*2-Methyl-2-(N-benzenesulfonyl-5-nitroindol-3-yl)-3-N-acetyl-5-phenyl-1,3,4-oxadiazolines* (**3s**): Yellow solid, m.p. 186–188 °C. ^1^H-NMR (400 MHz, CDCl_3_) δ: 8.42 (d, *J* = 2.0 Hz, 1H), 8.17–8.19 (m, 1H), 8.00–8.04 (m, 2H), 7.93–7.95 (m, 2H), 7.87–7.89 (m, 2H), 7.60–7.64 (m, 1H), 7.49–7.55 (m, 3H), 7.46 (t, *J* = 7.2 Hz, 2H), 2.36 (s, 3H), 2.33 (s, 3H). ^13^C-NMR (100 MHz, CDCl_3_) δ: 167.1, 154.0, 144.4, 137.9, 137.2, 134.7, 131.8, 129.7, 128.7, 128.4, 127.3, 127.0, 126.8, 124.0, 121.7, 120.1, 116.6, 113.9, 96.7, 23.6, 22.3. MS (ESI-TRAP), *m*/*z* (%): 527 ([M + Na]^+^, 100).

*2-Methyl-2-(N-benzenesulfonyl-6-methylindol-3-yl)-3-N-acetyl-5-phenyl-1,3,4-oxadiazolines* (**3y**): White solid, m.p. 142–144 °C. ^1^H-NMR (400 MHz, CDCl_3_) δ: 7.90 (d, *J* = 7.2 Hz, 2H), 7.86 (d, *J* = 7.2 Hz, 2H), 7.78 (s, 1H), 7.71 (s, 1H), 7.40–7.57 (m, 6H), 7.25–7.28 (m, 1H), 6.99 (d, *J* = 7.6 Hz, 1H), 2.40 (s, 3H), 2.31 (s, 3H), 2.27 (s, 3H). ^13^C-NMR (100 MHz, CDCl_3_) δ: 166.6, 153.9, 138.1, 135.6, 135.1, 133.9, 131.4, 129.3, 128.6, 126.8, 125.3, 124.6, 120.8, 119.4, 113.7, 97.2, 23.3, 22.2, 21.7. MS (ESI-TRAP), *m*/*z* (%): 496 ([M + Na]^+^, 100).

*2-Methyl-2-(N-benzenesulfonyl-5-cyanoindol-3-yl)-3-N-acetyl-5-phenyl-1,3,4-oxadiazolines* (**3z**): White solid, m.p. 208–210 °C. ^1^H-NMR (400 MHz, CDCl_3_) δ: 8.03 (d, *J* = 8.8 Hz, 1H), 7.97 (s, 1H), 7.94 (d, *J* = 7.6 Hz, 2H), 7.87 (d, *J* = 8.4 Hz, 2H), 7.75 (s, 1H), 7.60–7.64 (m, 1H), 7.51–7.56 (m, 4H), 7.43–7.47 (m, 2H), 2.31 (s, 6H). ^13^C-NMR (100 MHz, CDCl_3_) δ: 167.0, 153.9, 137.4, 136.8, 134.6, 131.8, 129.7, 128.7, 127.9, 127.8, 127.5, 127.0, 126.8, 125.0, 124.0, 120.8, 119.1, 114.5, 107.5, 96.7, 23.3, 22.2. MS (ESI-TRAP), *m*/*z* (%): 507 ([M + Na]^+^, 100).

*2-Methyl-2-(N-p-chlorobenzenesulfonyl-5-cyanoindol-3-yl)-3-N-acetyl-5-phenyl-1,3,4-oxadiazolines* (**3a′**)**:** White solid, m.p. 128–130 °C. ^1^H-NMR (400 MHz, CDCl_3_) δ: 8.01 (d, *J* = 8.8 Hz, 1H), 7.93 (s, 1H), 7.84–7.87 (m, 4H), 7.75 (s, 1H), 7.44–7.57 (m, 6H), 2.31 (s, 6H). ^13^C-NMR (100 MHz, CDCl_3_) δ: 167.0, 153.9, 141.5, 136.7, 135.7, 131.9, 130.0, 128.7, 128.3, 128.1, 127.7, 127.6, 126.8, 125.1, 123.9, 121.2, 118.9, 114.5, 107.7, 96.5, 23.3, 22.2. MS (ESI-TRAP), *m*/*z* (%): 541 ([M + Na]^+^, 100).

*2-Methyl-2-(N-p-methylbenzenesulfonyl-5-nitroindol-3-yl)-3-N-acetyl-5-phenyl-1,3,4-oxadiazolines* (**3b′**): Yellow solid, m.p. 110–112 °C. ^1^H-NMR (400 MHz, CDCl_3_) δ: 8.41 (d, *J* = 2.0 Hz, 1H), 8.16–8.19 (m, 1H), 7.99–8.02 (m, 2H), 7.89 (d, *J* = 7.2 Hz, 2H), 7.83 (d, *J* = 8.4 Hz, 2H), 7.42–7.52 (m, 3H), 7.32 (d, *J* = 8.0 Hz, 2H), 2.37 (s, 3H), 2.35 (s, 3H), 2.33 (s, 3H). ^13^C-NMR (100 MHz, CDCl_3_) *δ*: 167.1, 154.0, 146.1, 144.3, 137.8, 134.2, 131.8, 130.3, 128.7, 128.4, 127.2, 127.1, 126.8, 124.0, 121.5, 120.0, 116.6, 113.8, 96.8, 23.6, 22.3, 21.6. MS (ESI-TRAP), *m*/*z* (%): 541 ([M + Na]^+^, 100).

*2-Methyl-2-(N-p-chlorobenzenesulfonyl-5-nitroindol-3-yl)-3-N-acetyl-5-phenyl-1,3,4-oxadiazolines* (**3c′**)**:** Yellow solid, m.p. 108–110 °C. ^1^H-NMR (400 MHz, CDCl_3_) δ: 8.42 (d, *J* = 1.6 Hz, 1H), 8.18-8.21 (m, 1H), 7.97–8.02 (m, 2H), 7.85–7.89 (m, 4H), 7.43–7.52 (m, 5H), 2.35 (s, 3H), 2.33 (s, 3H). ^13^C-NMR (100 MHz, CDCl_3_) *δ*: 167.2, 154.0, 144.5, 141.6, 137.8, 135.6, 131.8, 130.1, 128.7, 128.4, 127.4, 126.9, 124.0, 122.1, 120.3, 116.8, 113.8, 96.6, 23.6, 22.3. MS (ESI-TRAP), *m*/*z* (%): 561 ([M + Na]^+^, 78).

*2-Ethyl-2-(N-benzenesulfonylindol-3-yl)-3-N-acetyl-5-phenyl-1,3,4-oxadiazolines* (**3d′**): White solid, m.p. 224–226 °C. ^1^H-NMR (400 MHz, CDCl_3_) δ: 7.85–7.93 (m, 6H), 7.41–7.56 (m, 7H), 7.25–7.29 (m, 1H), 7.13–7.17 (m, 1H), 3.05–3.11 (m, 1H), 2.54–2.59 (m, 1H), 2.31 (s, 3H), 1.03–1.07 (m, 3H). ^13^C-NMR (100 MHz, CDCl_3_) *δ*: 166.8, 154.5, 137.9, 135.1, 133.9, 131.5, 129.3, 128.6, 127.6, 126.9, 126.8, 125.8, 124.8, 124.3, 123.7, 120.9, 120.1, 113.7, 100.0, 28.4, 22.2, 6.5. MS (ESI-TRAP), *m*/*z* (%): 496 ([M + Na]^+^, 100).

### 3.3. Anti-HIV-1 Activity Assay

#### 3.3.1. Virus and Cells

Cell line (C8166) and the laboratory-derived virus (HIV-1_IIIB_) were obtained from MRC, AIDS Reagent Project, London, UK. C8166 was maintainedin RPMI-1640 supplemented with 10% heat-inactivated newborn calf serum (Gibco, Grand Island, NY, USA). The cells used in all experiments were in log-phase growth. The 50% HIV-1_IIIB_ tissue culture infectious dose (TCID_50_) in C8166 cells was determined and calculated by the Reed and Muench method. Virus stocks were stored in small aliquots at −70 °C.

#### 3.3.2. MTT-Based Cytotoxicity Assay

Cellular toxicity of 2-alkyl-2-(*N*-arylsulfonylindol-3-yl)-3-*N*-acyl-5-aryl-1,3,4-oxadiazolines **3s**–**r′** on C8166 cells was assessed by MTT method as described previously. Briefly, cells were seeded on 96-well microtiter plate in the absence or presence of various concentrations of 2-alkyl-2-(*N*-arylsulfonylindol-3-yl)-3-*N*-acyl-5-aryl-1,3,4-oxadiazolines in triplicate and incubated at 37 °C in a humid atmosphere of 5% CO_2_ for 3 day. The supernatants were discarded and MTT reagent (5 mg/mL in PBS) was added to each wells, then incubated for 4 h, 100 μL of 50% *N*,*N*-dimethylformamide (DMF)-20% SDS was added. After the formazan was dissolved completely, the plates were read on a Bio-TekElx800 ELISA reader (BioTek, Winooski, VT, USA) at 595/630 nm. The cytotoxic concentration that caused the reduction of viable C8166 cells by 50% (CC_50_) was determined from dose–response curve.

#### 3.3.3. Syncytia Assay

In the presence of 100 μL various concentrations of 2-alkyl-2-(*N*-arylsulfonylindol-3-yl)-3-*N*-acyl-5-aryl-1,3,4-oxadiazolines, C8166 cells (4 × 10^5^/mL) were infected with virus HIV-1_IIIB_ at a multiplicity of infection (M.O.I) of 0.06. The final volume per well was 200 μL. Control assays were performed without the testing compounds in HIV-1_IIIB_ infected and uninfected cultures. After 3 days of culture, the cytopathic effect (CPE) was measured by counting the number of syncytia. Percentage inhibition of syncytia formation was calculated and 50% effective concentration (EC_50_) was calculated. AZT (Sigma-Aldrich, St. Louis, MO, USA) was used as a positive control. Therapeutic index (TI) = CC_50_/EC_50_.

## 4. Conclusions

Here we report a very superior method of the microwave-assisted expeditious synthesis of 2-alkyl-2-(*N*-arylsulfonylindol-3-yl)-3-*N*-acyl-5-aryl-1,3,4-oxadiazolines catalyzed by HgCl_2_ under solvent-free conditions. This method has the advantages of low catalyst loading and recovering catalyst, short reaction and repaid reaction times, easy separation products, excellent yields, and being more conducive to the large-scale synthesis products. Compound **3i′** especially displayed the highest anti-HIV-1 activity with TI values of 39.59. It implied that **3i′** might be regarded as the lead compound for further preparation of anti-HIV-1 agents.

## Figures and Tables

**Table 1:**
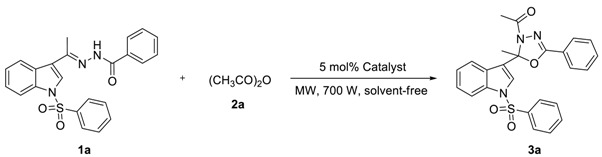
Screening of the catalyst for the cyclization reaction between **1a** and **2a**.

Entry	Amount of (mmol)		Catalyst (5 mol%)	*t*^a^ (min)	Isolated Yield ^b^ (%)	
	1a	2a			3a	1a
1	0.5	1.5	MgCl_2_**^.^**6H_2_O	10 × 3	38	37
2	0.5	1.5	ZnCl_2_	10 × 3	48	23
3	0.5	1.5	AlCl_3_	10 × 3	53	9
4	0.5	1.5	SnCl_2_**^.^**2H_2_O	10 × 3	63	15
5	0.5	1.5	FeCl_3_	10 × 3	68	18
6	0.5	1.5	HgCl_2_	10 × 3	91	0

^a^ 10 × 3 means three times 10 min as reaction time, and the progress of the reaction was checked by TLC analysis at the end of each irradiation period. ^b^ Isolated yield (%) after preparative thin-layer chromatography.

**Table 2:**
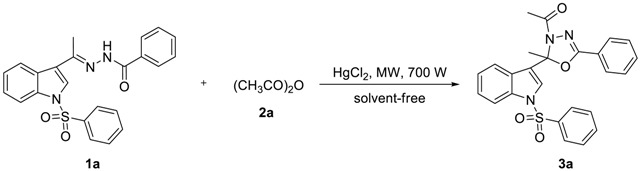
Optimization of the reaction conditions.

Entry	Amount of (mmol)		HgCl_2_ (mol%)	*t*^a^ (min)	Isolated Yield ^b^ (%)	
	1a	2a			3a	1a
1	0.5	2.5	5	10 × 2	97	0
2	0.5	2.0	5	10 × 2	90	0
3	0.5	1.5	5	10 × 3	91	0
4	0.5	1.0	5	10 × 4	41	55
5	0.5	2.5	0	10 × 12	0	100
6	0.5	2.5	2.5	10 × 5	69	26
7	0.5	2.5	3	10 × 3	78	17
8	0.5	2.5	4	10 × 3	91	0
9	0.5	1.5	4	10 × 3	90	0

^a^ 10 × 2 means two times 10 min as reaction time, and the progress of the reaction was checked by TLC analysis at the end of each irradiation period. ^b^ Isolated yield (%) after preparative thin-layer chromatography.

**Table 3:**
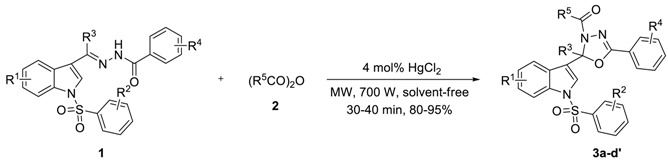
Synthesis of 2-alkyl-2-(*N*-arylsulfonylindol-3-yl)-3-*N*-acyl-5-aryl-1,3,4-oxadiazolines (**3a**–**d′**) ^a^.

3a–d′	1				2	*t*^b^ (min)	Yield ^c^ (%)
	R^1^	R^2^	R^3^	R^4^	R^5^		
**3a**	H	H	Me	H	Me	10 × 3	90
**3b**	H	*p*-Me	Me	H	Me	10 × 3	90
**3c**	6-Me	*p*-Me	Me	H	Me	10 × 3	95
**3d**	H	*p*-Cl	Me	H	Me	10 × 4	87
**3e**	6-Me	*p*-Cl	Me	H	Me	10 × 4	90
**3f**	H	*p*-Cl, *m*-NO_2_	Me	H	Me	10 × 4	90
**3g**	6-Me	*p*-Cl, *m*-NO_2_	Me	H	Me	10 × 4	89
**3h**	H	*m*-NO_2_	Me	H	Me	10 × 4	85
**3i**	6-Me	*m*-NO_2_	Me	H	Me	10 × 4	84
**3j**	5-CN	*m*-NO_2_	Me	H	Me	10 × 4	81
**3k**	5-CN	*p*-Me	Me	H	Me	10 × 4	84
**3l**	H	*p*-OMe	Me	H	Me	10 × 3	90
**3m**	6-Me	*p*-OMe	Me	H	Me	10 × 3	93
**3n**	H	*p*-Me	Et	H	Me	10 × 3	87
**3o**	H	H	*n*-Pr	H	Me	10 × 3	80
**3p**	H	*p*-Me	*n*-Pr	H	Me	10 × 3	86
**3c**	H	*p*-Cl	Et	H	Me	10 × 3	88
**3r**	H	H	Me	*m*-Me	Me	10 × 3	84
**3s**	H	*p*-Me	Me	*m*-Me	Me	10 × 3	87
**3t**	6-Me	*m*-NO_2_	Me	H	Et	10 × 3	81
**3u**	H	*p*-Me	Et	H	Et	10 × 3	88
**3v**	H	*p*-Me	*n*-Pr	H	Et	10 × 3	87
**3w**	H	H	Me	*m*-Me	Et	10 × 3	85
**3s**	5-NO_2_	H	Me	H	Me	10 × 4	90
**3y**	6-Me	H	Me	H	Me	10 × 3	81
**3z**	5-CN	H	Me	H	Me	10 × 4	83
**3a′**	5-CN	*p*-Cl	Me	H	Me	10 × 4	80
**3b′**	5-NO_2_	*p*-Me	Me	H	Me	10 × 4	87
**3c′**	5-NO_2_	*p*-Cl	Me	H	Me	10 × 4	88
**3d′**	H	H	Et	H	Me	10 × 3	82

^a^ A mixture of **1** (0.5 mmol), **2** (1.5 mmol), and HgCl_2_ (0.02 mmol) was reacted under microwave heating at 700 W for 30–40 min. ^b^ 10 × 3 means three times 10 min as reaction time, and the progress of the reaction was checked by TLC analysis at the end of each irradiation period. ^c^ Isolated yield (%) after preparative thin-layer chromatography.

**Table 4:**
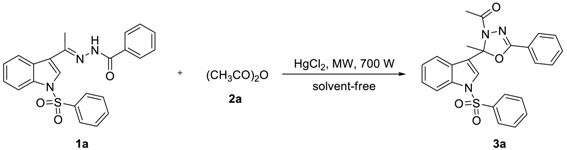
Typical procedure for recovering HgCl_2_ catalyst.

Entry	Amount of (mmol)		HgCl_2_ (mol%)	*t* (min)	Isolated Yield ^a^ (%)
	1a	2a			3a
1	3	9	4	5	98
2	3	9	4	6	98
3	3	9	4	8	97
4	3	9	4	9	98

^a^ Isolated yield (%) after preparative thin-layer chromatography.

**Table 5:** Anti-HIV-1 activity of 2-alkyl-2-(*N*-arylsulfonylindol-3-yl)-3-*N*-acyl-5-aryl-1,3,4-oxadiazolines **3s**–**r′** in vitro ^a^.

3s–r′	CC_50_^b^ (μg/mL)	EC_50_^c^ (μg/mL)	TI ^d^
**3s**	109.42	3.35	32.66
**3y**	>200	6.12	>32.68
**3z**	53.03	17.57	3.01
**3a′**	113	3.63	31.22
**3b′**	132.88	9.54	13.94
**3c′**	53.4	6.01	8.89
**3d′**	105.21	20.33	5.18
**3e′**	15.09	15.52	0.97
**3f′**	43.45	1.79	24.27
**3g′**	80.79	22.48	3.59
**3h′**	4.8	3.53	1.36
**3i′**	20.19	0.51	39.59
**3j′**	2.26	12.42	0.18
**3k′**	16.57	19.37	0.86
**3l′**	181.84	57.49	3.16
**3m′**	12.33	22.48	0.55
**3n′**	11.19	16.56	0.68
**3o′**	151.65	18.14	8.36
**3p′**	134.98	63.8	2.12
**3q′**	78.04	3.00	26.01
**3r′**	98.31	4.01	24.51
**AZT ^e^**	1373.17	0.00199	690,035

^a^ Values are means of two separate experiments. ^b^ CC_50_ (50% cytotoxic concentration), concentration of drug that causes 50% reduction in total C8166 cell number. ^c^ EC_50_ (50% effective concentration), concentration of drug that reduces syncytia formation by 50%. ^d^ In vitro therapeutic index (CC_50_ value/EC_50_ value). ^e^ AZT was used as a positive control.

## Supplementary Materials

The following are available online. The NMRs and MS of the new compounds (**3s**–**d′**).
