# Supplementary material for: Microwave-Assisted Expeditious Synthesis of 2-Alkyl-2-(N-arylsulfonylindol-3-yl)-3-N-acyl-5-aryl-1,3,4-oxadiazolines Catalyzed by HgCl2 under Solvent-Free Conditions as Potential Anti-HIV-1 Agents
Source: Molecules. 2018 Nov 10;23(11):2936. doi: 10.3390/molecules23112936 (PMC6278315; doi:10.3390/molecules23112936)

Supporting Information

Microwave-Assisted Expeditious Synthesis of 2-Alkyl-2-(*N*-Arylsulfonylindol-3-yl)-3-*N*-Acyl-5-Aryl-1,3,4-Oxadiazolines Catalyzed by $HgCl_2$ under Solvent-Free Conditions as potential Anti-HIV-1 Agents

Zhiping Che[#]*, Yuee Tian[#], Shengming Liu, Jia Jiang, Mei Hu and Genqiang Chen

Laboratory of Pharmaceutical Design & Synthesis, Department of Plant Protection, College of Forestry, Henan University of Science and Technology, Luoyang 471003, Henan Province, People′s Republic of China

*Corresponding author. Tel./fax: +86-0379-64389603. E-mail address: zhipingche@163.com (Z. P. Che).

[#]These authors contributed equally to this work.

CONTENTS

Copies of ^{1}H, ^{13}C NMR, and MS spectra of compounds 3s-d'.

3s. ^{1}H NMR

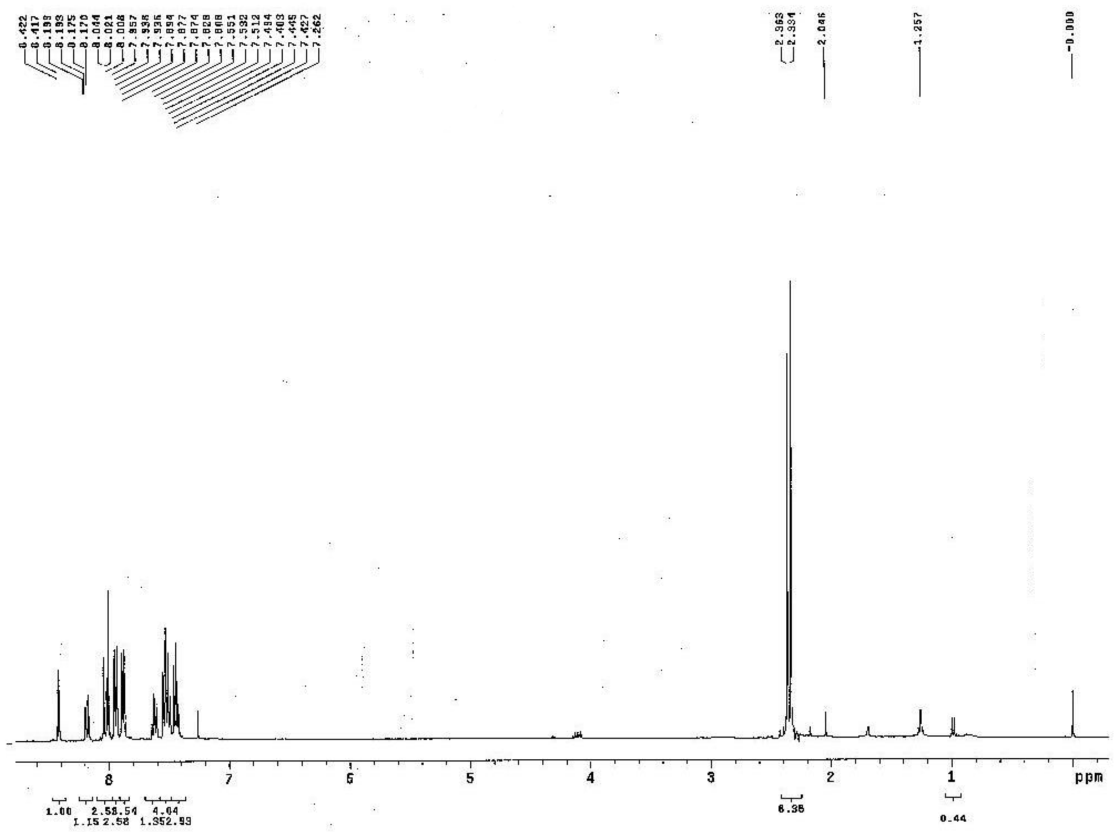

3s. ^{13}C NMR

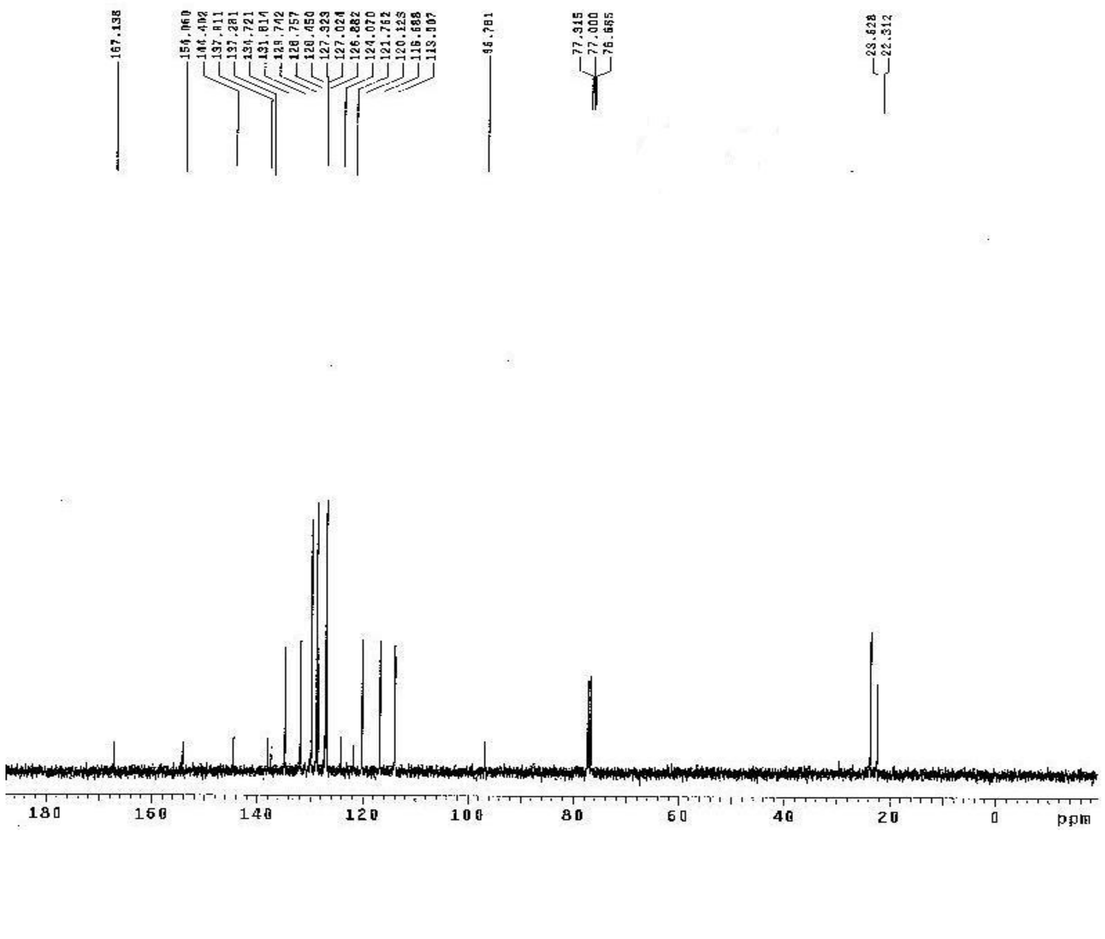

3s. MS (ESI-TRAP)

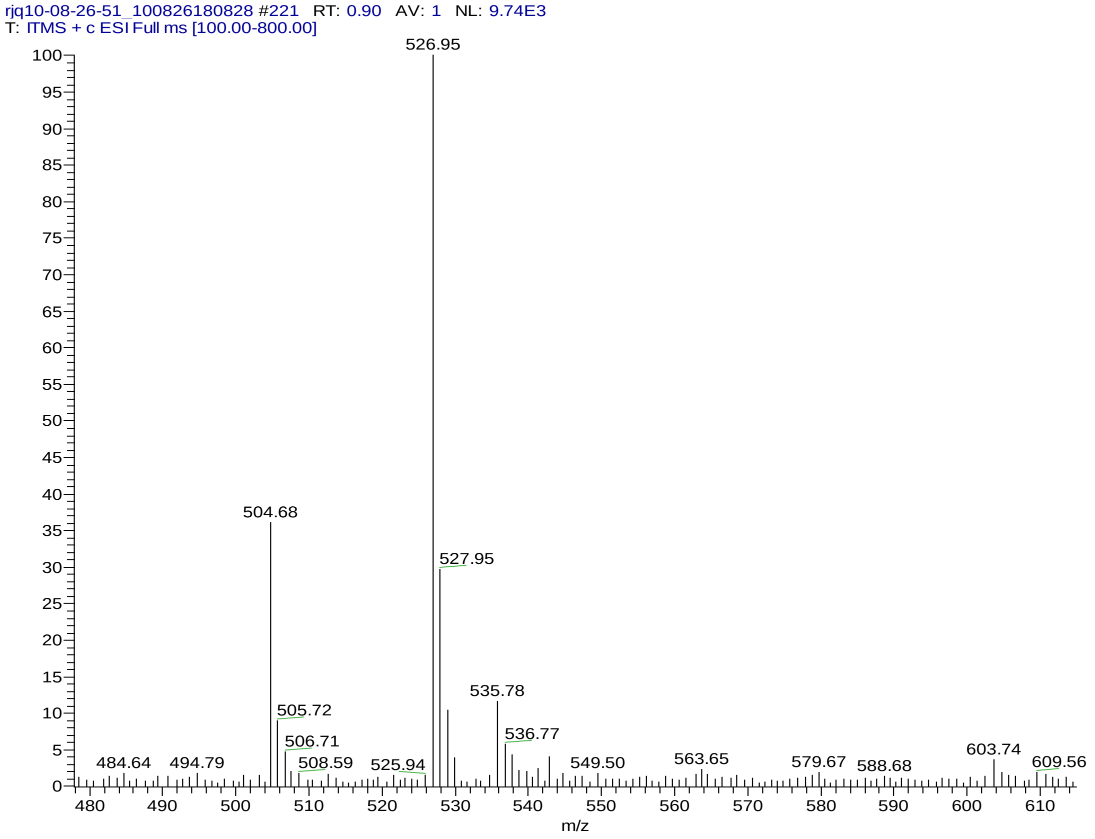

3y. ^{1}H NMR

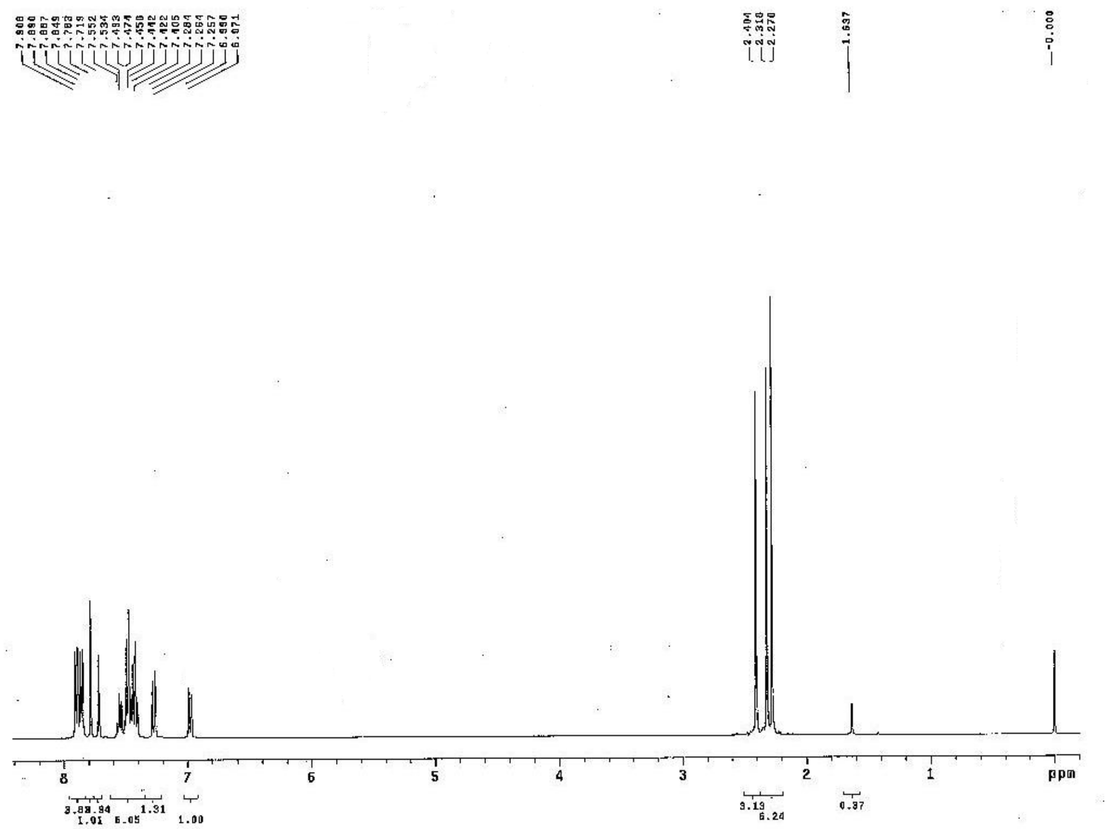

3y. ^{13}C NMR

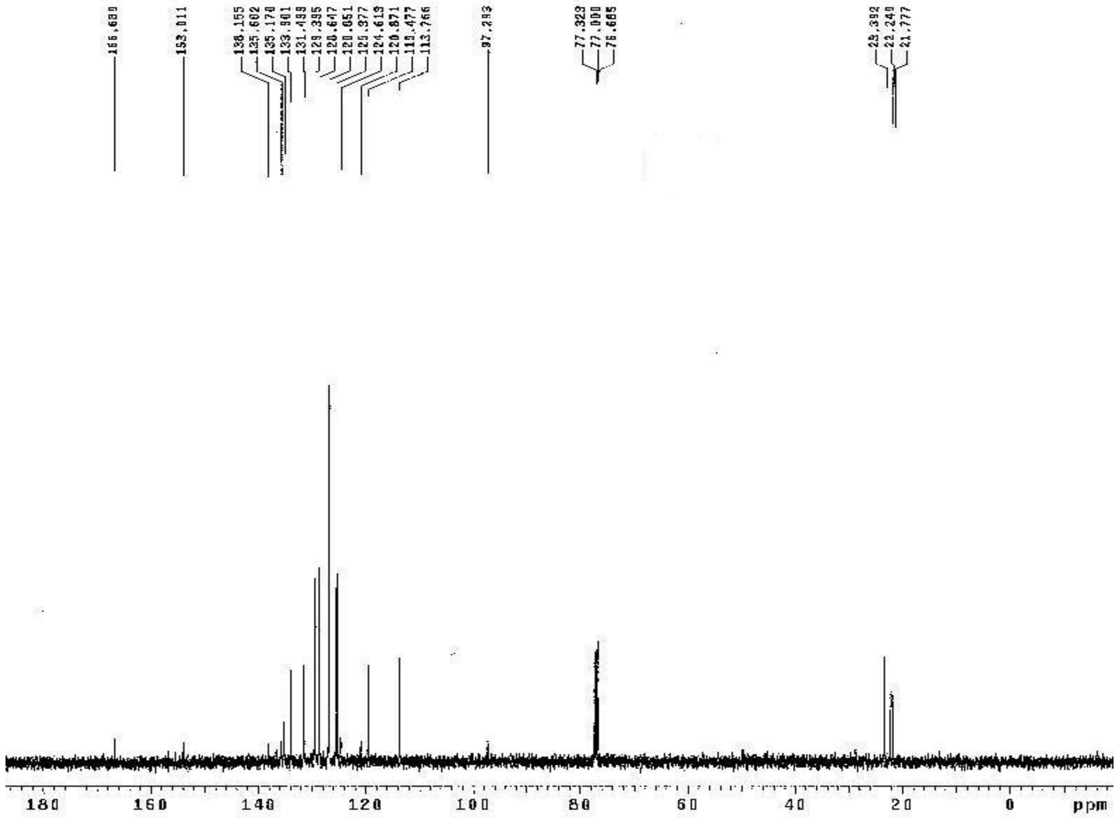

3y. MS (ESI-TRAP)

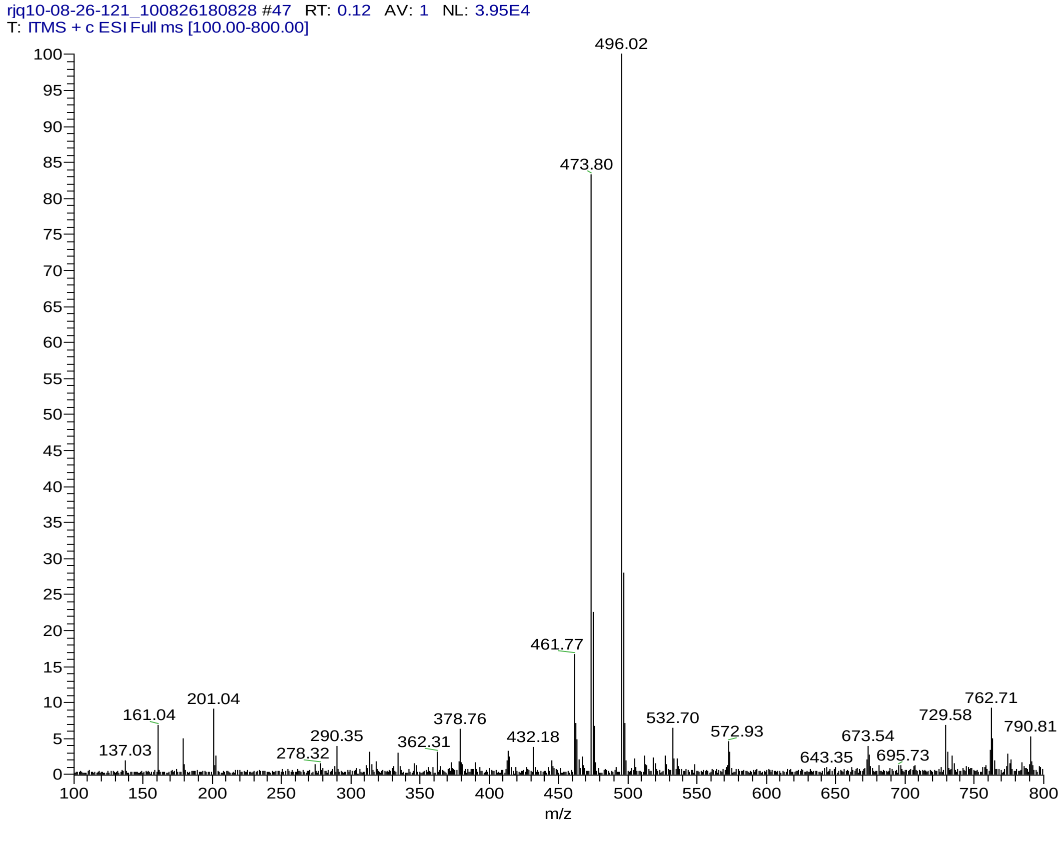

3z. ^{1}H NMR

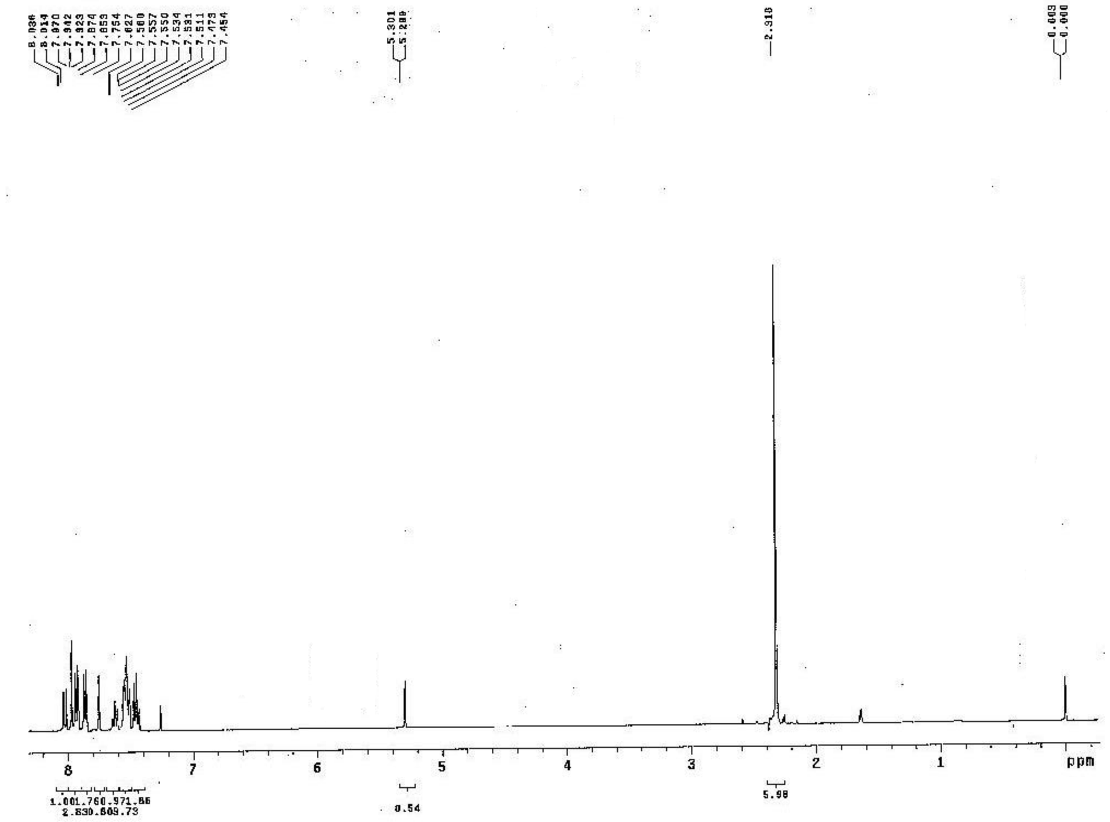

3z. ^{13}C NMR

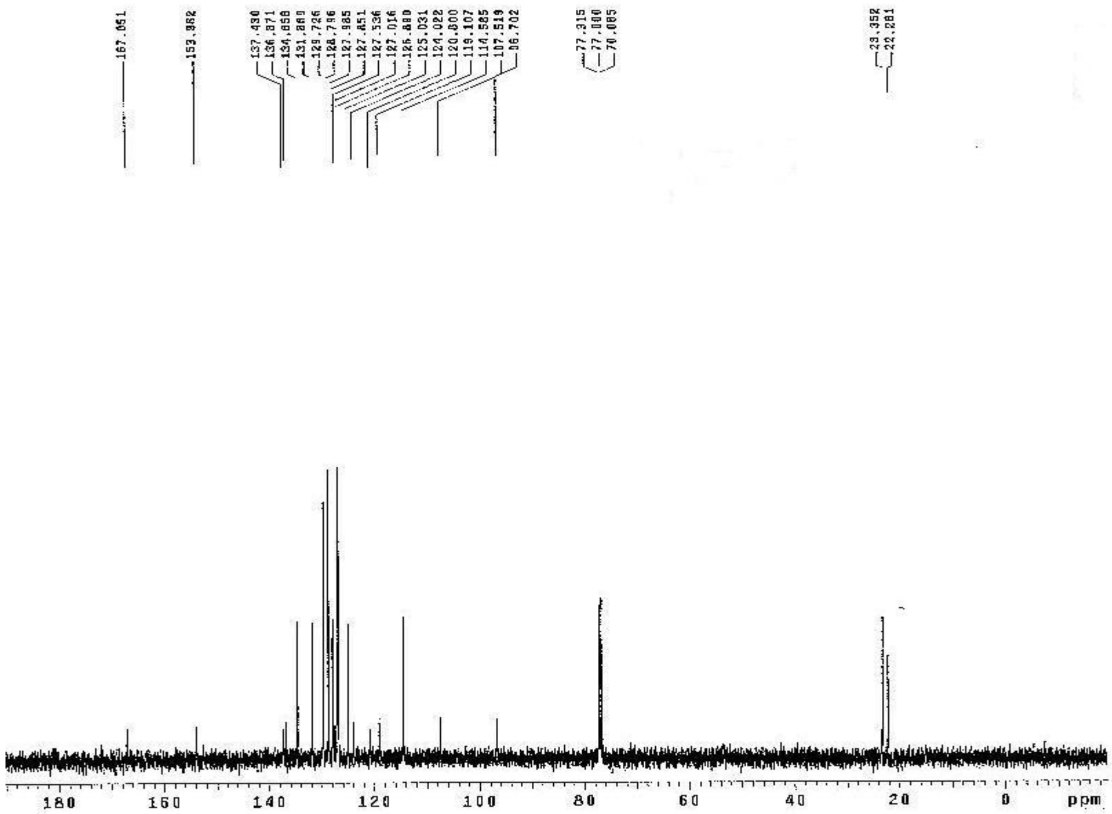

3z. MS (ESI-TRAP)

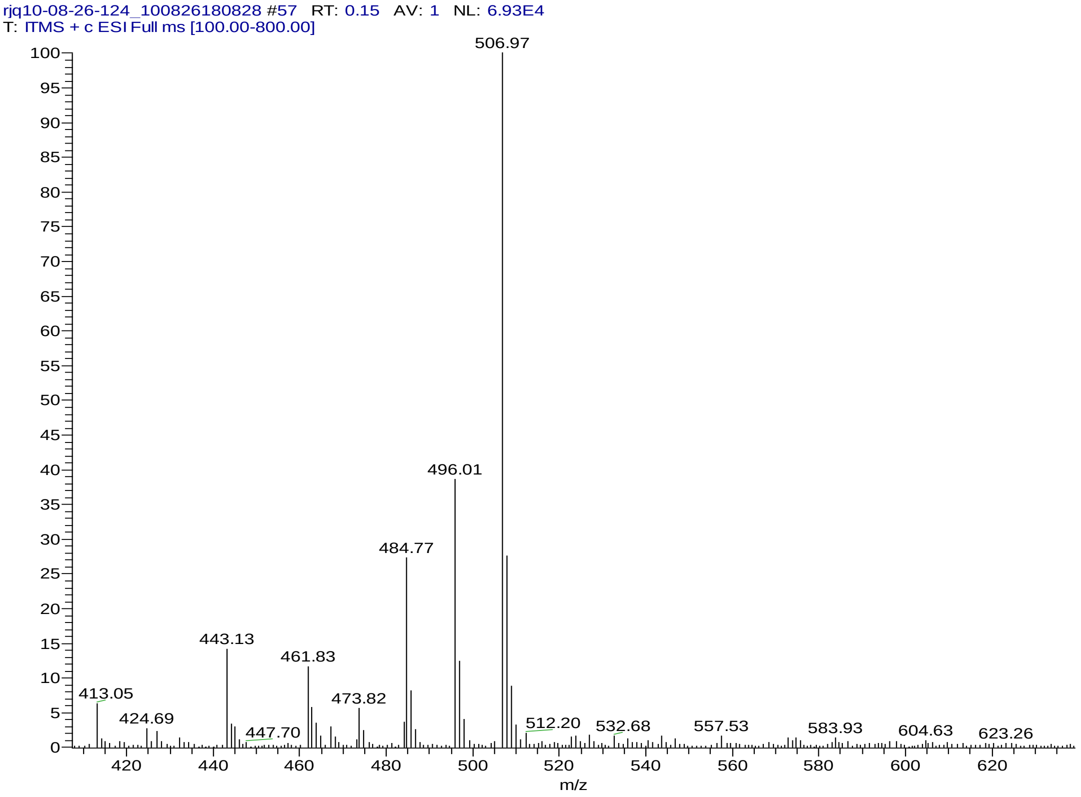

3a'. ^{1}H NMR

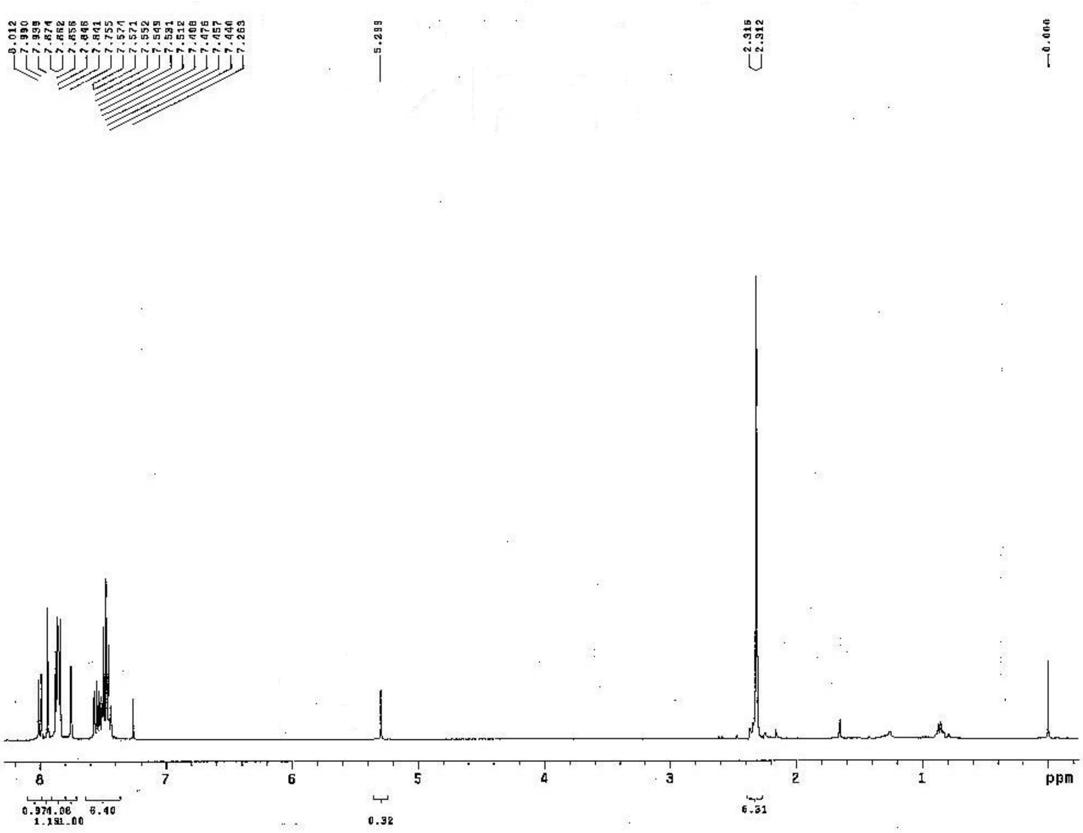

3a'. ^{13}C NMR

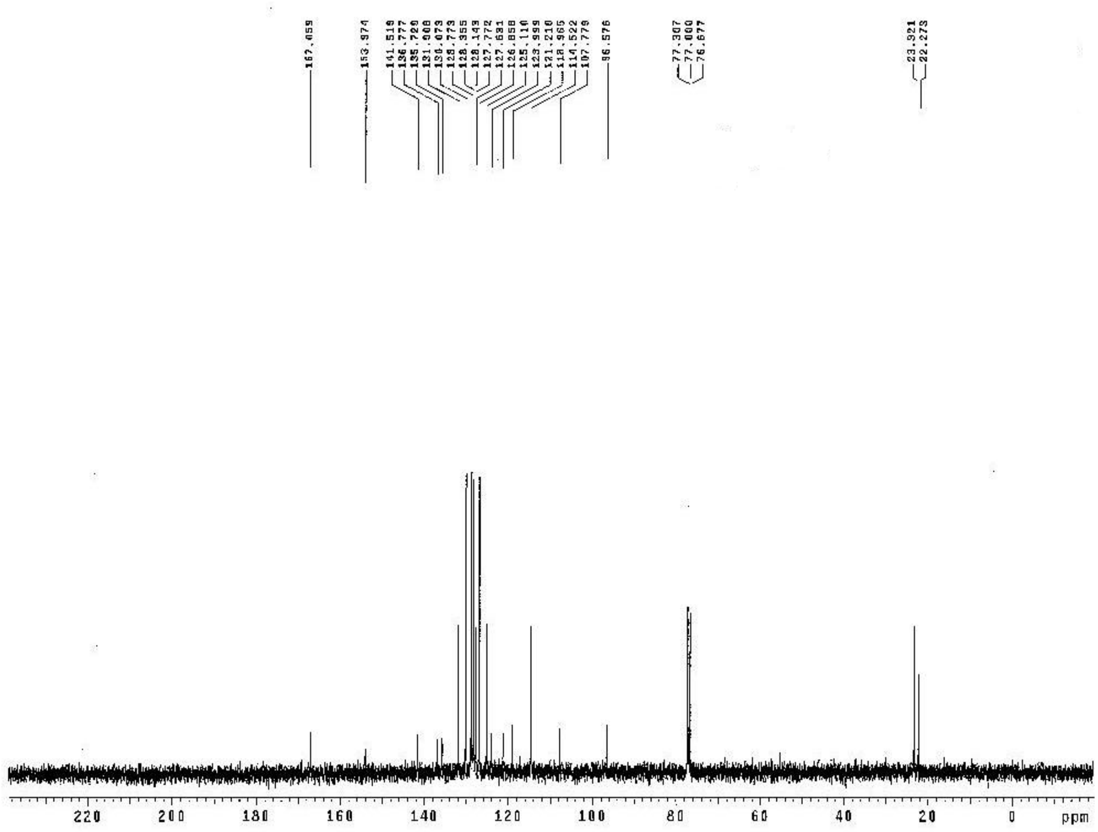

3a'. MS (ESI-TRAP)

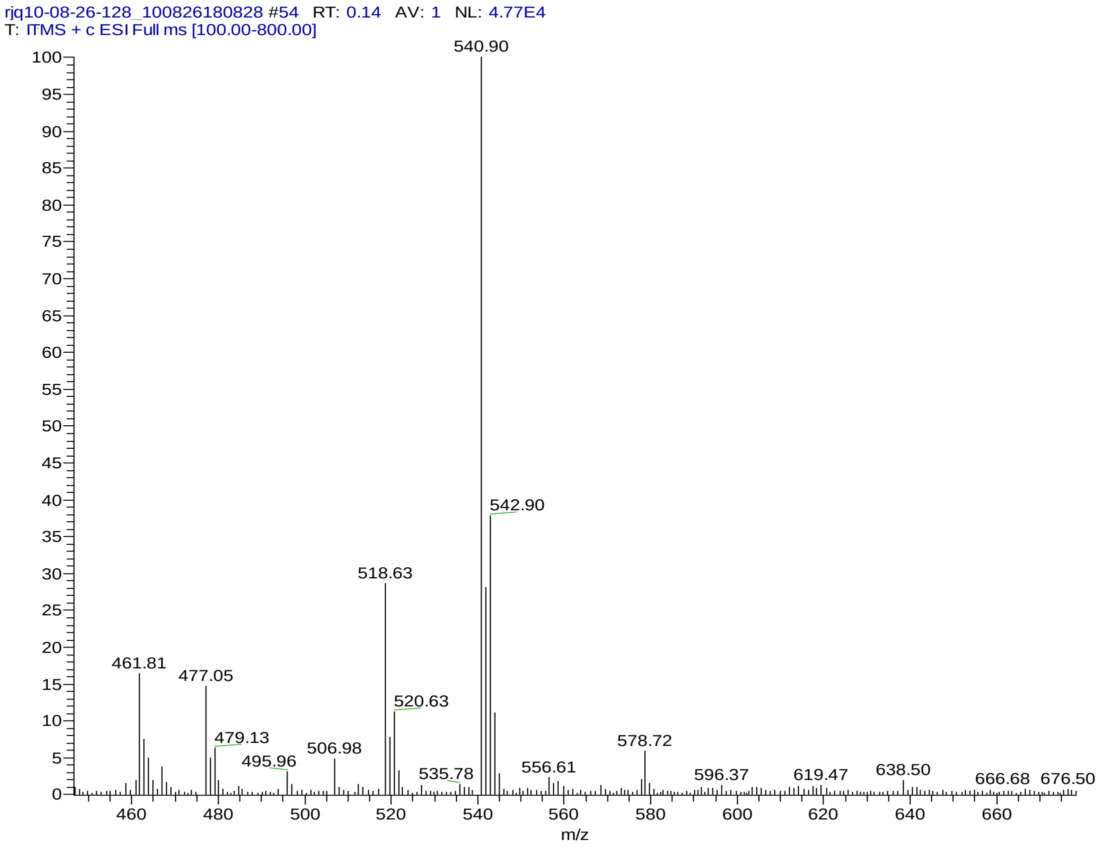

3b'. ^{1}H NMR

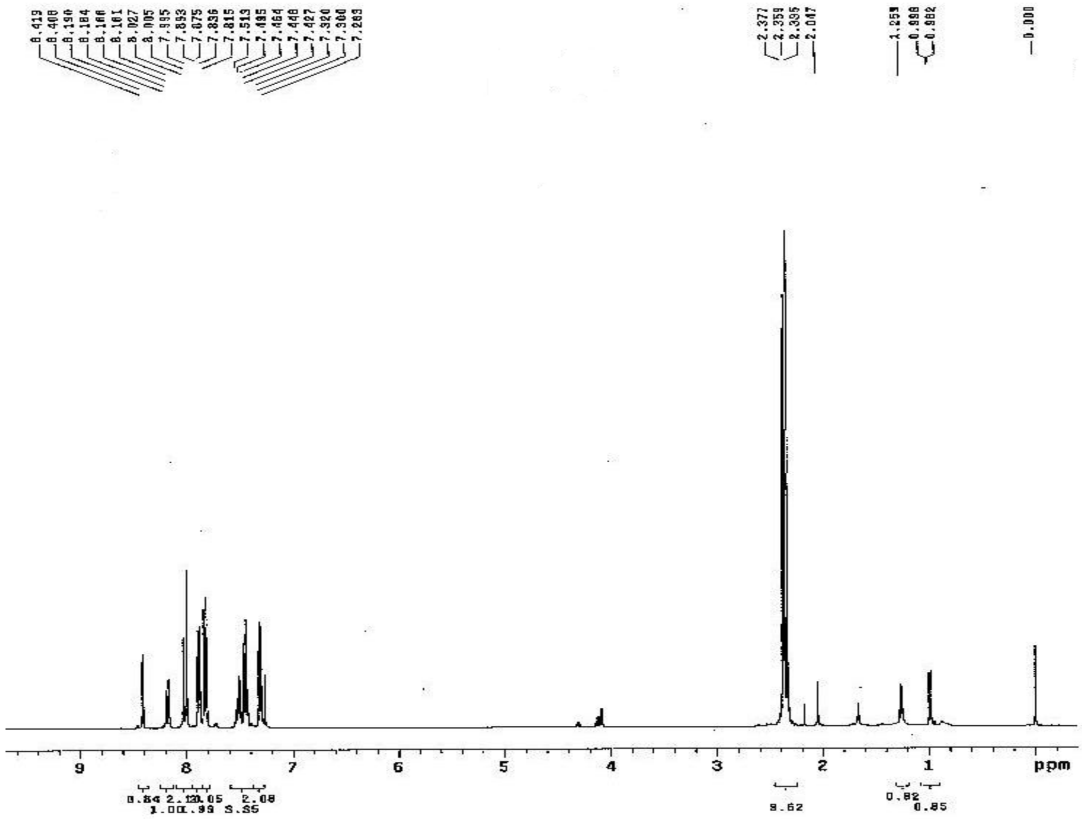

3b'. ^{13}C NMR

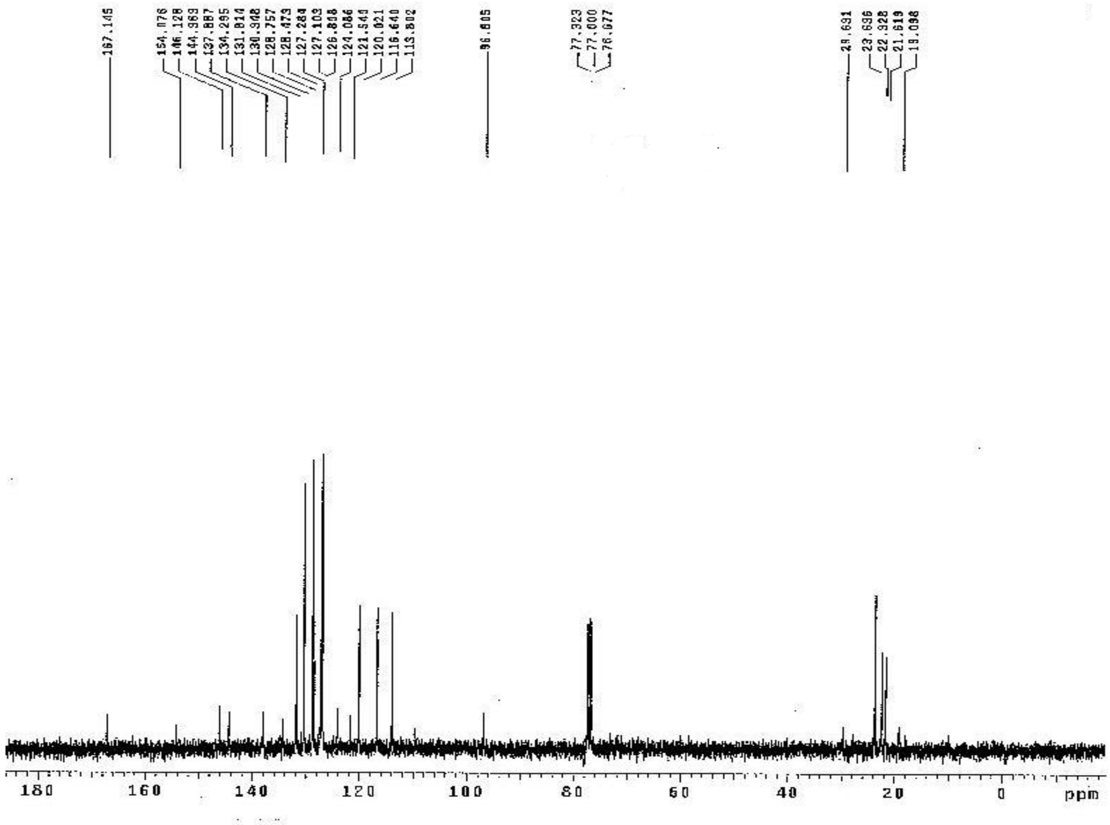

3b'. MS (ESI-TRAP)

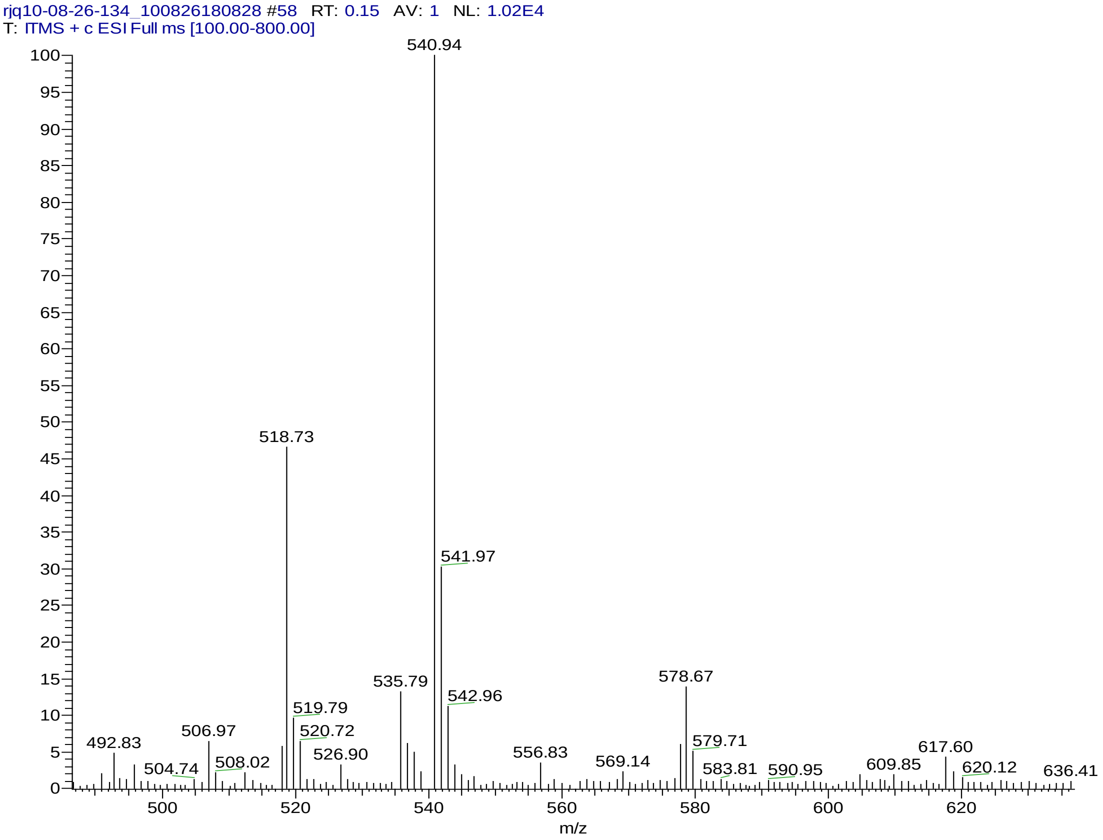

3c'. ^{1}H NMR

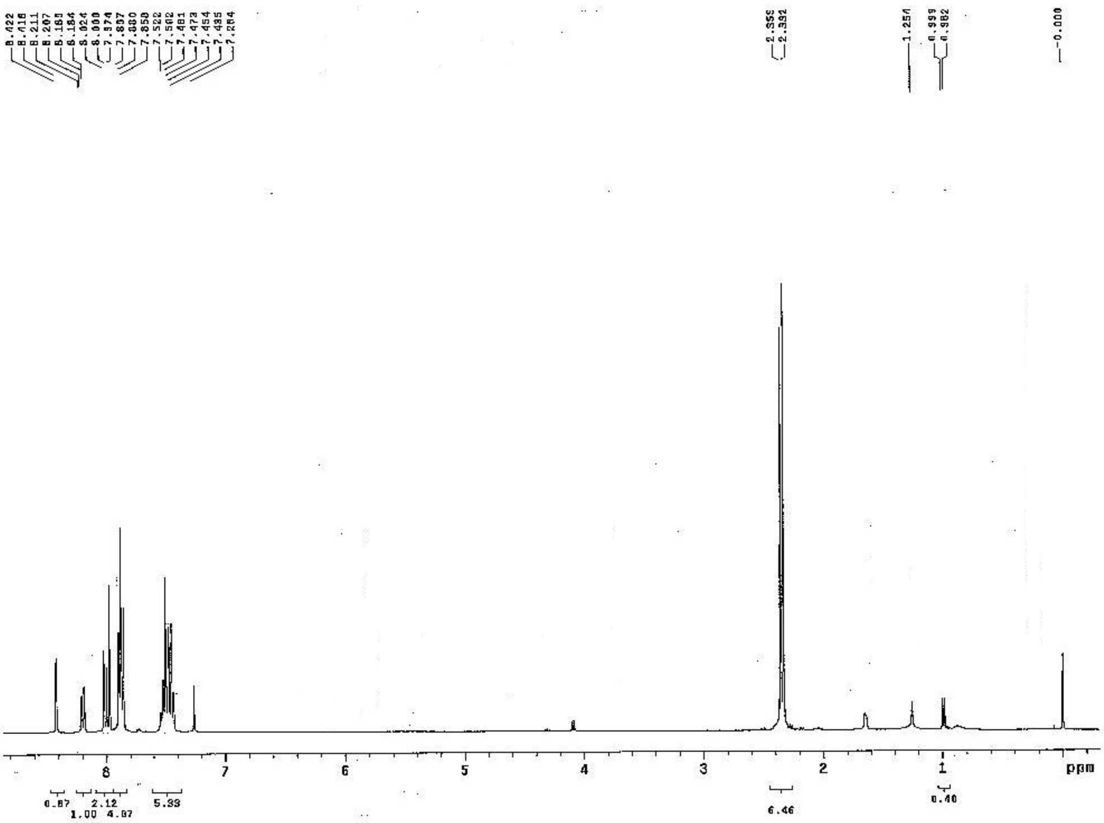

3c'. ^{13}C NMR

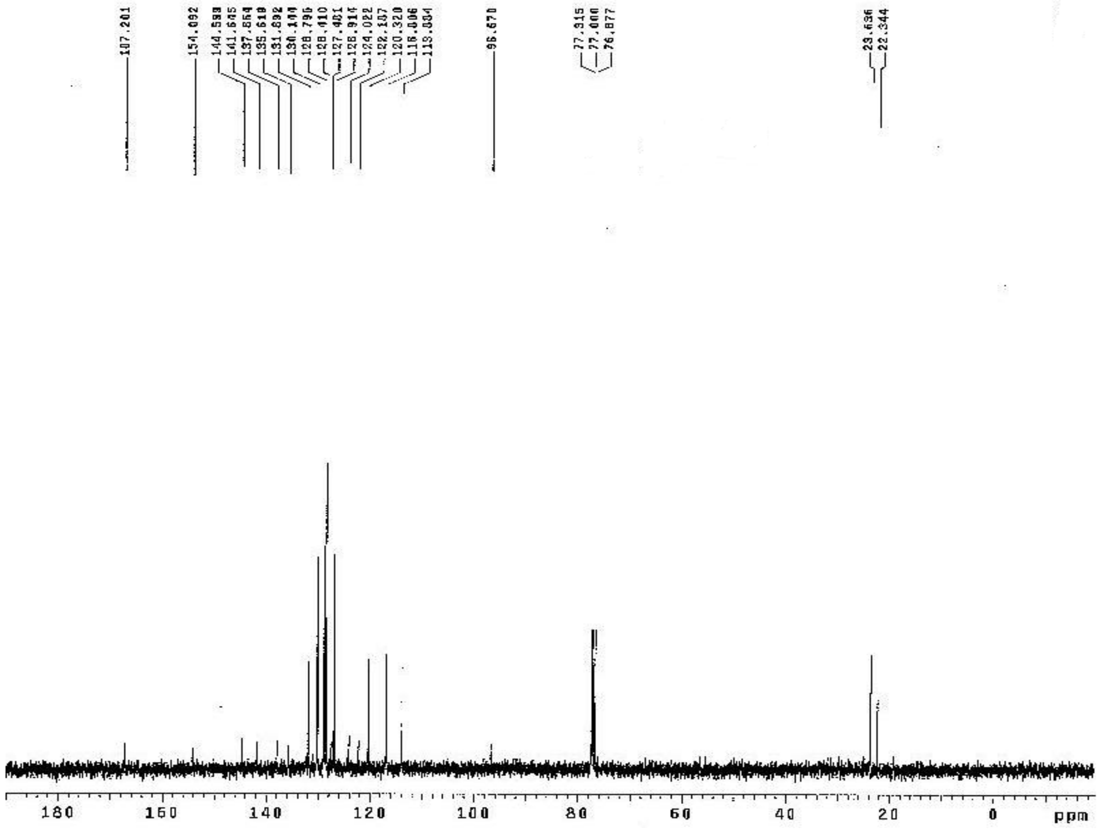

3c'. MS (ESI-TRAP)

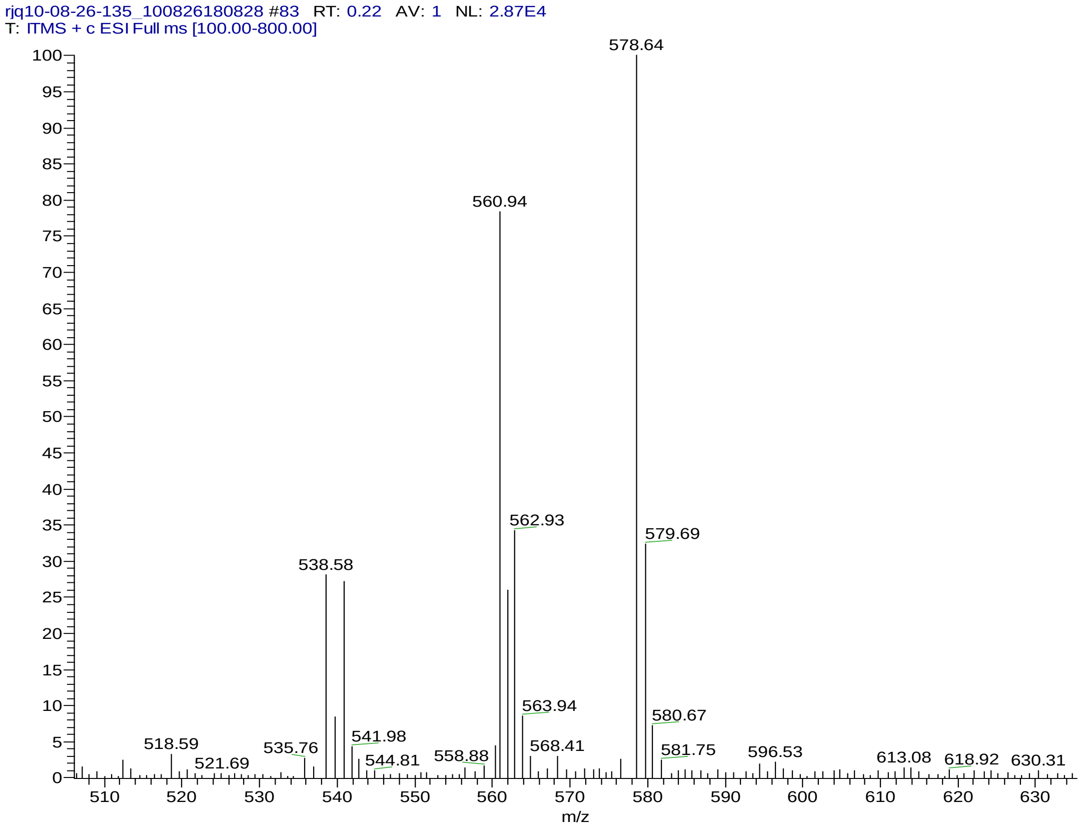

3d'. ^{1}H NMR

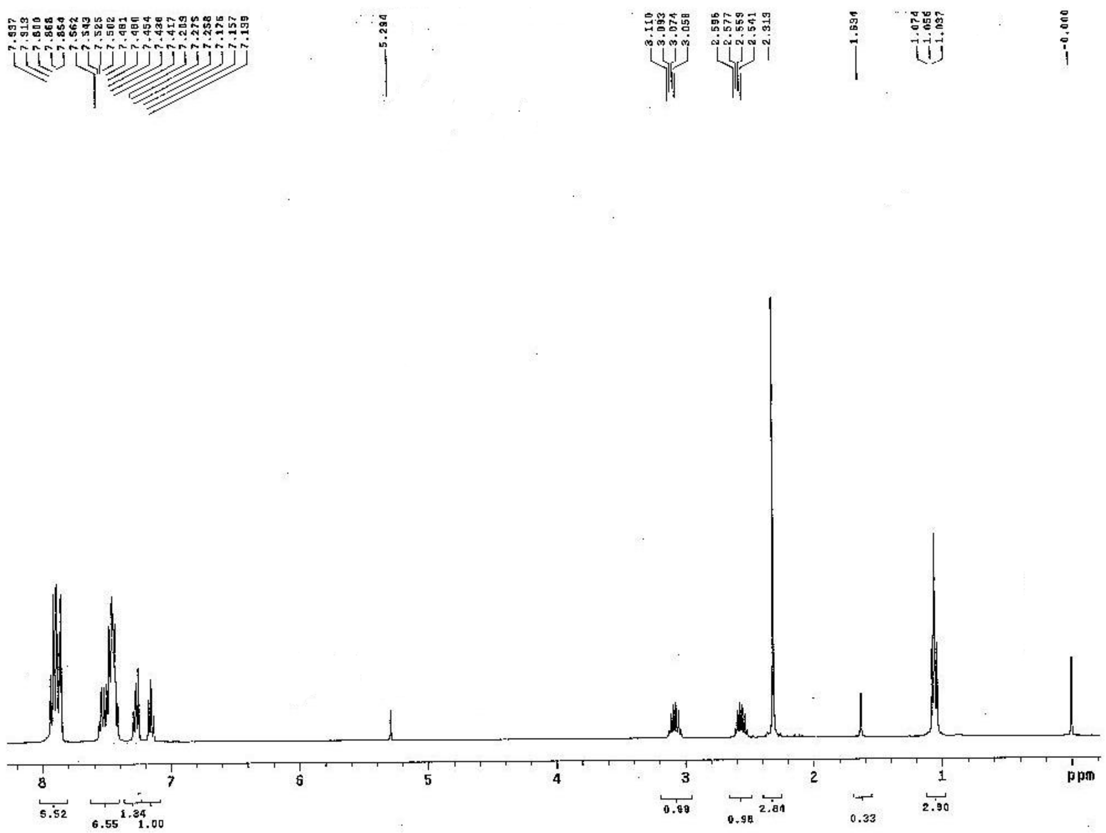

3d'. ^{13}C NMR

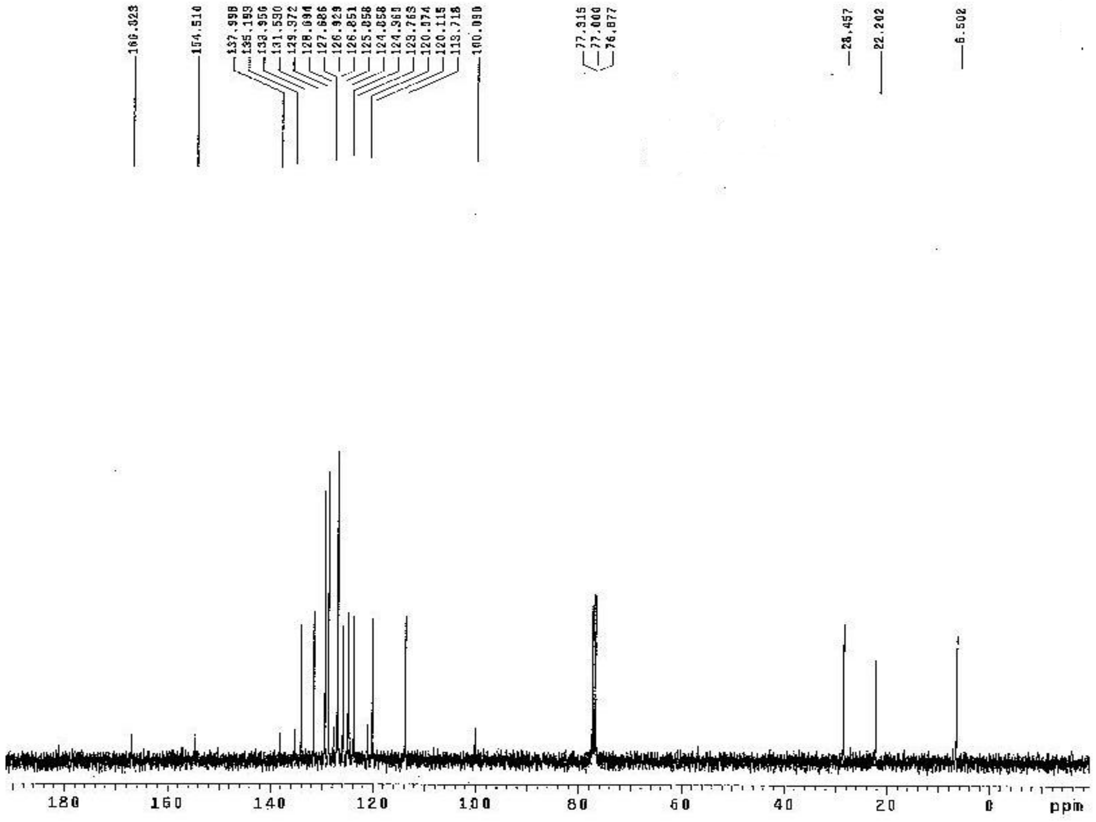

3d'. MS (ESI-TRAP)

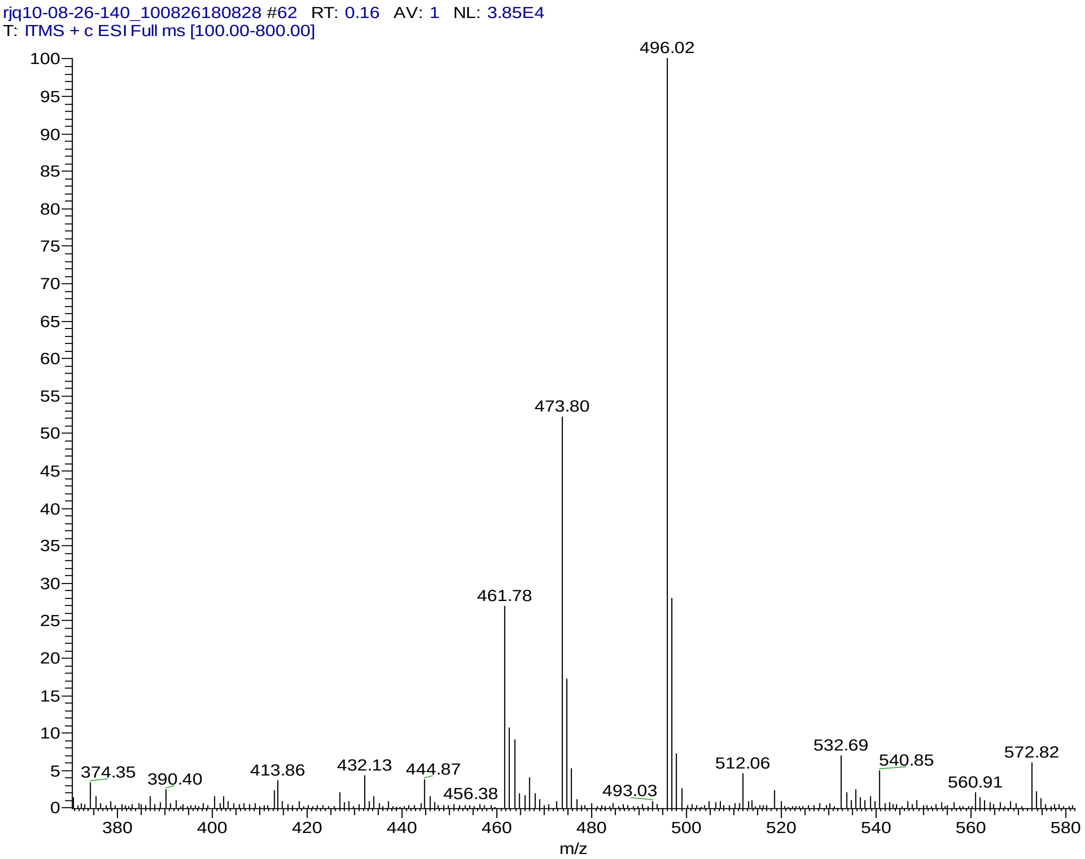

Supplement: Supplementary file 1 [file molecules-23-02936-s001.pdf]
